# SPECT/CT 90Y-Bremsstrahlung images for dosimetry during therapy

**DOI:** 10.3332/ecancer.2008.106

**Published:** 2008-11-11

**Authors:** C Fabbri, G Sarti, M Agostini, A Di Dia, G Paganelli

**Affiliations:** 1 Division of Medical Physics, Ospedale Bufalini, Cesena, Italy; 2 Division of Nuclear Medicine, Ospedale Bufalini, Cesena, Italy; 3 Division of Nuclear Medicine, Istituto Europeo di Oncologia, Via Ripamonti 435, 20141 Milano, Italy

## Abstract

**Background:**

the characteristics of ^90^Y, suitable for therapy, are denoted by the lack of γ-emission. Alternative methods, using analogues labelled with ^111^In or ^86^Y, are generally applied to image ^90^Y-conjugates, with some inevitable drawbacks. New generation SPECT/CT image systems offer improved Bremsstrahlung images. The intent of this brief communication is to show that high quality ^90^Y-Bremsstrahlung SPECT-CT images can be obtained, allowing the biodistribution of pure β-emitter therapeutical agents to be evaluated, also during the course of therapy.

**Methods:**

the hybrid system Siemens Symbia-T2 was used for the acquisition of images of a patient given 1.7 GBq of ^90^Y-DOTATATE. The following parameters were set for SPECT: 80 (50%) and 120 (30%) keV energy windows; medium energy collimators; 128 × 128 matrix, 64 projections (40s/step). Low-dose CT was acquired (80 mAs) for attenuation correction. Images were reconstructed with the OSEM 3D-Fast algorithm.

**Results:**

post-therapy SPECT-CT ^90^Y-Bremsstrahlung images of a patient undergoing receptor peptide radionuclide therapy are presented. ^90^Y-Bremsstrahlung images obtained are suitable for tumour and normal organ dosimetry, providing detailed information on biodistribution, comparable to ^111^In-diagnostic images.

**Conclusions:**

the improved Bremsstrahlung images means that the diagnostic examinations can be used for patient recruitment and that dosimetry evaluation can be restricted only to treated patients. This could avoid the need for a different radionuclide or isotope to mimic therapy. The clinical impact might be notable, as dosimetry and toxicity information are essential in radionuclide therapy, especially in patients with risk factors.

A 53-year old man, diabetic, with an unresectable endocrine pancreatic carcinoma was diagnosed with additional abdominal metastases as documented by CT and biopsy. In August 2007, scintigraphic images with octreoscan demonstrated high uptake in the abdominal lesions. Figure 1A shows the trans-axial and coronal slices of fused SPECT-CT, with uptake in the abdominal lesion (red arrow), kidneys and spleen. In November 2007, the patient underwent the first cycle of receptor peptide radionuclide therapy with 1.7 GBq of ^90^Y-DOTATATE.

Post-therapy SPECT-CT ^90^Y-Bremsstrahlung images were acquired at 24 and 48 hours, with the same settings, excluding energy windows, as for octreoscan. The hybrid system Siemens Symbia T2 was used for SPECT acquisition, with medium energy collimators, 80 (50%) and 120 (30%) keV energy windows, 128 × 128 matrix, 64 projections (40 s/step). Low-dose CT was acquired (80 mAs) and used for attenuation correction. Images were reconstructed with the OSEM 3D Fast algorithm with automatic correction for 3D response of the collimator.

The ^90^Y-Bremsstrahlung images obtained give more detailed information on biodistribution, in comparison to the ^111^In-diagnostic images, which are more suitable for tumour and normal organ dosimetry. Figure 1B shows the corresponding trans-axial and coronal slices of fused SPECT-CT as with octreoscan. Increased uptake in the abdominal lesion (red arrow) and higher uptake in the renal cortex than the medulla (yellow arrow) [1] can be clearly distinguished. This is consistent with the different distribution of DOTATATE versus octreoscan.

The higher quality of the Bremsstrahlung images means that the diagnostic examination can be aimed at patient recruitment (suitability or not for treatment) and so restricting dosimetry evaluation only to treated patients. This could avoid the need for a tracer to mimic therapy and overcome difficulties of ^86^Y quantification [2]. The clinical impact might be notable as dosimetry and toxicity information are essential in radionuclide therapy especially in patients with associated risk factors (e.g. diabetes) [3].

## Figures and Tables

**Figure 1:**
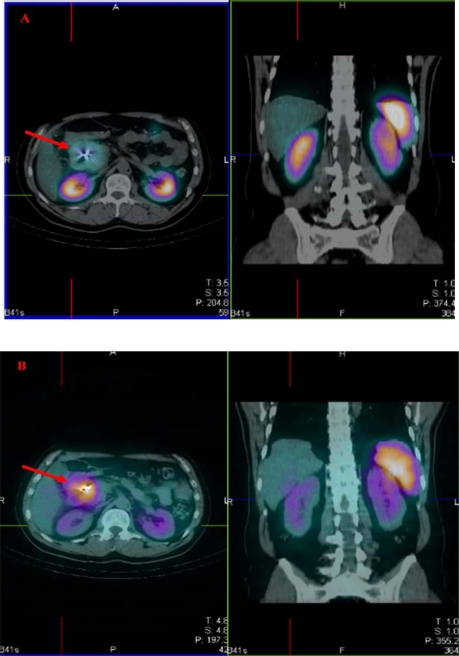
Trans-axial and coronal slices of fused SPECT-CT, showing uptake in the abdominal lesion (red arrow), the kidneys (yellow arrow) and the spleen. Images obtained 24 h after the injection of (A) octreoscan (185 MBq), (B) ^90^Y-DOTATATE (1.7 GBq)
